# Occupations of Great Men

**Published:** 1859-06

**Authors:** 


### OCCUPATIONS OF GREAT MEN.

Trousseau, one of the most able and eminent of living physicians says, he brought on an asthma by measuring oats one night, to see whether his coachman stole them. How could one of the greatest medical minds of the age find time to measure oats for the purpose of "diagnosing" the honesty of his carriage driver; it reminds us of our drawing "our four" in a dollar slide through Irving Place last winter. How they delighted to be spilt out in the snow. A day or two earlier, there passed our window, one of the oldest and most eminent and respected divines of our city eating a piece of veritable ginger cake with the most don't care look possible.

It is said that Napoleon the first was found by one of his ministers of state crawling on all fours, with his child riding on his back, endeavoring now and then to brush him off by going under the table. These cases show that great men have no notion of being always on stilts; they get tired of living in the clouds, and come down now and then for recreation, to measure oats, sleigh ride children, and munch gingerbread; and why not, if they chose to do so!

